# Clinical, imaging and histopathological characterization of a series of three cats with cerebellar cortical degeneration

**DOI:** 10.1186/s12917-024-04127-3

**Published:** 2024-06-19

**Authors:** Céline Giron, Pierre Hélie, Joane Parent, Mathieu Boutin, Guillaume St-Jean

**Affiliations:** 1 Department of clinical sciences, Faculty of veterinary medicine, Université de Montréal, Saint-Hyacinthe, QC J2S 2M2 Canada; 2 Department of pathology and microbiology, Faculty of veterinary medicine, Université de Montréal, Saint-Hyacinthe, QC J2S 2M2 Canada

**Keywords:** Purkinje cells, Cerebellar cortical degeneration, Genetic, MRI, Inherited

## Abstract

**Background:**

Neurological inherited disorders are rare in domestic animals. Cerebellar cortical degeneration remains amongst the most common of these disorders. The condition is defined as the premature loss of fully differentiated cerebellar components due to genetic or metabolic defects. It has been studied in dogs and cats, and various genetic defects and diagnostic tests (including magnetic resonance imaging (MRI)) have been refined in these species. Cases in cats remain rare and mostly individual, and few diagnostic criteria, other than post-mortem exam, have been evaluated in reports with multiple cases. Here, we report three feline cases of cerebellar cortical degeneration with detailed clinical, diagnostic imaging and post-mortem findings.

**Case presentation:**

The three cases were directly (siblings, case #1 and #2) or indirectly related (same farm, case #3) and showed early-onset of the disease, with clinical signs including cerebellar ataxia and tremors. Brain MRI was highly suggestive of cerebellar cortical degeneration on all three cases. The relative cerebrospinal fluid (CSF) space, relative cerebellum size, brainstem: cerebellum area ratio, and cerebellum: total brain area ratio, were measured and compared to a control group of cats and reference cut-offs for dogs in the literature. For the relative cerebellum size and cerebellum: total brain area ratio, all affected cases had a lower value than the control group. For the relative CSF space and brainstem: cerebellum area ratio, the more affected cases (#2 and #3) had higher values than the control group, while the least affected case (#3) had values within the ranges of the control group, but a progression was visible over time. Post-mortem examination confirmed the diagnosis of cerebellar cortical degeneration, with marked to complete loss of Purkinje cells and associated granular layer depletion and proliferation of *Bergmann glia*. One case also had Wallerian-like degeneration in the spinal cord, suggestive of spinocerebellar degeneration.

**Conclusion:**

Our report further supports a potential genetic component for the disease in cats. For the MRI examination, the relative cerebellum size and cerebellum: total brain area ratio seem promising, but further studies are needed to establish specific feline cut-offs. Post-mortem evaluation of the cerebellum remains the gold standard for the final diagnosis.

## Background

Inherited neurological disorders are rare in domestic animals, especially in cats [1]. Most described inherited diseases in cats belong to the large family of storage diseases, including gangliosidosis [2], neuronal ceroid lipofuscinosis [3], etc. Diseases affecting the development of the cerebellum, although relatively frequent in cats, are infrequently inherited disorders. For example, cerebellar hypoplasia, one of the most common neurological disease in cats, is a well described condition in neonatal and young kittens infected *in utero* or postnatally with feline panleukopenia virus (parvovirus) [4]. Aside from developmental cerebellar conditions, diseases affecting primarily the cerebellum are rare in cats. In infectious conditions such as in toxoplasmosis and feline infectious peritonitis, the resulting meningoencephalitis may affect the cerebellum, but the involvement is rather diffuse [5, 6]. However, there are some reports of potentially inherited cerebellar cortical degeneration [7, 8].

Cerebellar cortical degeneration, often referred as cerebellar cortical abiotrophy, is considered among the most common neurodegenerative disease in domestic animals [9]. It is defined as the premature degeneration of fully differentiated cerebellar cells, most frequently the Purkinje cells, due to genetic or metabolic defects [10]. The condition has been described in a wide range of domestic animals [11], including dogs [12–18], horses [19–23], goats [24], sheep [25], rabbits [26], cows [27] and cats [7, 8, 28–31]. Although the genetic etiology and diagnostic venues have been extensively investigated in dogs and horses [32, 33], few diagnostic criteria, other than post-mortem, have been evaluated in the rare series of feline cases.

For this report, we present a series of three feline cases of cerebellar cortical degeneration with detailed clinical, diagnostic imaging and post-mortem findings, with comparative analysis of criteria reported in other species (dogs and horses). We also further support the precedent reports suggesting a potential genetic etiology of the disease, as two of the cases were direct siblings, whereas the third was born on the same farm (‘barn cats’) within a few years’ gap. This raises the question of a potential genetically inheritable defect present in this farm’s feline population.

## Case presentation

### Clinical presentation

#### CASE #1

A domestic shorthair cat, intact female, was presented to the neurology department of the Centre Hospitalier Universitaire Vétérinaire of the Université de Montréal (CHUV) at 7 months of age for evaluation of abnormal gait following the examination of a littermate (Case #2) with similar symptoms. At time of adoption, she had always lived outdoors. The owner thus could not tell when the clinical signs had appeared. When adopted, the cat was in good body condition, except for a severe Toxocara infection. In addition, the cat had lost the tips of its ears due to cold weather exposure. She was FIV/FeLV seronegative. On neurological examination, there was cerebellar ataxia involving all limbs. The cat was visual. The menace response was absent on the left and reduced on the right. In addition, the physiological nystagmus was slower on the right. During the induction of the physiological nystagmus, the animal took an abnormal posture, curling on itself, suspected to be a vestibular posture. Moreover, the induced horizontal nystagmus persisted transiently following circling the cat from right to left.

At 9 months of age, magnetic resonance imaging (MRI) of the brain was performed and the animal was neutered. On re-evaluation at 10 months, the cat was clinically stable and ambulatory with cerebellar ataxia and occasional fine intention tremors. The menace responses were bilaterally absent. There was exaggerated head and neck extension when physiological nystagmus was assessed.

At 15 months of age, the owner reported deterioration in the cat’s neurological condition. There was worsening of the cerebellar ataxia with frequent stumbling and falling. The animal was sitting on his pelvic limbs and had a hunched posture. The menace responses were decreased to absent bilaterally. A nystagmus persisted following head movements.

At 16 months of age, brain MRI was repeated with owner’s permission, and the cat was euthanized following the procedure. A complete necropsy was performed.

#### CASE #2

A domestic shorthair cat, male neutered, was presented to the CHUV at 6.5 months of age. Since his adoption at 6 weeks of age from a farm, it presented an abnormal gait and tremors. Two out of five kittens from the same litter (including case #1) were similarly affected. When presented to a veterinarian at 2.5 months of age, mild cerebellar signs were suspected. At presentation to the CHUV, the owner reported the intention tremors to be stable, but the gait abnormalities had worsened. On examination, there was cerebellar ataxia with hypermetria and spasticity in all limbs. The hopping reactions were delayed with exaggerated responses. The menace responses were absent bilaterally.

Two months later, the owner mentioned that the neurological condition had worsened; the cat fell more often and did not jump on furniture anymore. The tremors were unchanged. The owner reported that back discomfort may be present and felt that its tail was not normal. On neurological examination, there was moderate to severe cerebellar ataxia characterized by hypermetria and spasticity in all limbs. Intention tremors were also present. The cat was visual. There was an absence of menace responses bilaterally.

Re-evaluated 1.5 years later (2 years old), the owner described his gait as stable. The cat fell as much as before and remained unable to climb on furniture. Also, he seemed to move more slowly. For the past few weeks, the cat had been defecating outside of his litter box, about two feet away from it. He had had two episodes of rhinitis in the past year treated with antibiotics. At that time, he was still living with two cats, and interacted well. On neurological examination, the cat meowed loudly when held. There were severe cerebellar ataxia and presence of intention tremors. There was a lack of menace responses bilaterally. The physiological nystagmus was absent. Head and neck carriage was abnormal with spastic ventro-flexion. Hopping was decreased in the thoracic limbs and absent in the pelvic limbs. Muscle tone was increased in all limbs.

At 3 years of age, the cat was presented to the emergency department for dysuria. A diagnosis of feline urologic syndrome was made. Over the following 24 h, there was urethral blockage necessitating more invasive procedures. Considering the patient’s neurological status, euthanasia was elected. With owner’s permission, brain MRI was made prior to euthanasia and necropsy was granted for educational purpose.

#### CASE #3

A domestic shorthair cat, male neutered, was presented to the CHUV at 18 months of age. He was presented for euthanasia due to suspected cerebellar ataxia.

The cat had been adopted at 2 months of age from the same farm where cases #1 and #2 originated. At time of adoption, the animal was already walking diagonally, but had no obvious tremors. Gradually, he started showing loss of balance. The clinical signs became more pronounced around 4 months of age. Tremors were more marked, and the cat started to fall when jumping on furniture. He was presented to a first veterinarian who performed blood work with no abnormalities. The cat’s clinical signs continued to worsen. At about 6 months of age, he was evaluated by a neurologist. The cat walked only for short distances. He walked along the walls. He presented moderate, relatively symmetrical cerebellar ataxia with a vestibular component more marked on the right. Intention tremors involving the entire body were observed. No menace response was present on the right. Brain MRI was proposed but declined. Empirical treatment with antibiotic (clindamycin) and corticosteroidwas initiated to exclude an inflammatory cause (infectious and non-infectious). Treatment was stopped after two weeks due to lack of response.

The gait continued to worsen. The cat’s quality of life was markedly impaired as the tremors were continuous, especially at feeding time.

The cat had been neutered and had received his basic vaccines and deworming. He was FIV/FELV seronegative. He was a strictly indoor cat and lived with another cat. The cat had no recent health problems.

Given the severity of the clinical signs, and the lack of response to treatment, euthanasia was decided upon. Post-mortem brain MRI was performed with owner’s permission. A necropsy followed.

## Summary of the three cases

Although the severity of the clinical signs varied among the cats, the clinical signs were characteristic of cerebellar involvement in all three cats. The mental status was appropriate, the gait was hypermetric, the tremors were intentional, the decreased to absent menace responses related to the cerebellum (the cats were visual and had palpebral reflexes) and the abnormalities in the nystagmus were associated to the vestibular part of the cerebellum.

At time of examination of the first cat, cerebellar cortical degeneration was suspected. The progression of the clinical signs in these cats ruled out in utero or perinatal infection with feline panleukopenia virus. Neuronal storage diseases were ruled out as the deterioration of the clinical signs remained associated to the cerebellum. Cats with neuroaxonal dystrophy have abnormal coat color and the lesions involve the brainstem as well as the cerebellum [34].

Due to the similar conclusion of all three cases, the MRI and pathology observations are presented grouped in the future sections.

A summary of the different clinical findings for the three cases can be found in Table 1.

**Table 1:** Summary of neurological presentation of each cat. NR = not reported

	Cerebellar Ataxia	Tremors	Tonus	Menace response	Physiological Nystagmus
Case #1	++	+	NR	Decreased (bilateral)	Present
Case #2	+++	++	↑	Absent (bilateral)	Absent
Case #3	++	++	NR	Absent (right)	NR

### MRI interpretation

Images of the brain of all cases were acquired with a 1.5 Tesla magnet (GE Signa EchoSpeed HDx; GE Healthcare, Chicago, Illinois, USA) using a knee coil. All cases had at minimum the following sequences: transverse, sagittal and dorsal T2-weighted (T2w) fast spin echo (FSE), and transverse TI-weighted (T1w) FSE or T1w fluid attenuated inversion recovery (FLAIR). Cases #1 and #2 had also T2w FLAIR and T2w single shot fast spin echo (SSFSE) images. Transverse and dorsal or sagittal T1w FLAIR images were acquired following IV injection of gadobenate dimeglumine (Multihance 529 mg/mL ®; Bracco Imaging Canada, Anjou, Quebec), at 0.1 mmol/kg for cases #1 and #2.

**Fig. 1:**
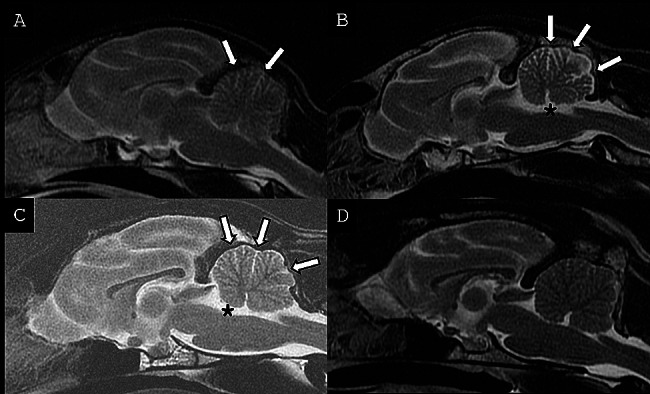
Mid-sagittal T2w FSE MRI images of the brain of three cats diagnosed with cerebellar cortical degeneration. (**A**) Case #1 at time of diagnosis, (**B**) Case #2, (**C**) Case #3 and **D**) unaffected feline brain for comparison. In the three cases, there is increased conspicuity of CSF space between cerebellar folia (arrows) of varying severity, considered subjectively moderate to marked in B) and C), and mild in A). There is also a mild distension of the fourth ventricle in B) and (C) (asterix)

**Fig. 2:**
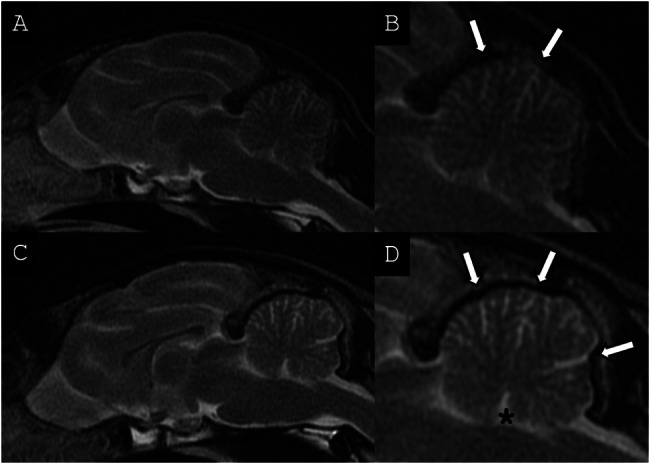
Mid-sagittal T2w FSE MRI images of the brain of case #1. (**A**) At time of imaging diagnosis with (**B**) close-up on the cerebellum. (**C**) Follow up 8 months later with (**D**) close-up on the cerebellum. There is moderate progression over time of the increased conspicuity of CSF space between cerebellar folia within both examinations (white arrows). The cerebellum has mildly decreased size and a mild progressive distension of the fourth ventricle is also noted at follow-up (asterix)

All cases presented cerebellar abnormalities, with a subjectively decreased cerebellar size with apparent increased cerebrospinal fluid (CSF) space between the cerebellar folia. These changes were associated with a variably enlarged fourth ventricle. These changes are illustrated in Fig. 1 (A-C), and compared with an unaffected feline patient (D), presented for non-cerebellar clinical signs. The MRI findings were more marked at presentation in cases #2 and #3 as compared to case #1 as illustrated in Fig. 1. Case #1 had two brain MRI examinations in an 8 month-period, as presented in Fig. 2. At his follow-up examination, there was a subjective progression of the increased CSF space between the cerebellar folia, consistent with progressive cerebellar cortical degeneration. No other MRI abnormalities were noted on the brain of the three cats. For cases #1 and #2, no evidence of abnormal enhancement was noted on the T1w post contrast images. For all three cases, the primary differential diagnosis was cerebellar cortical degeneration. Another less likely differential diagnosis for all three cases was a congenital disease such as cerebellar hypoplasia, possibly associated to an in utero or perinatal infection such as feline panleukopenia virus (parvovirus). It was considered unlikely due to the progressive nature of the neurological signs during adulthood. A lysosomal storage disease was considered unlikely considering the imaging changes.

**Fig. 3:**
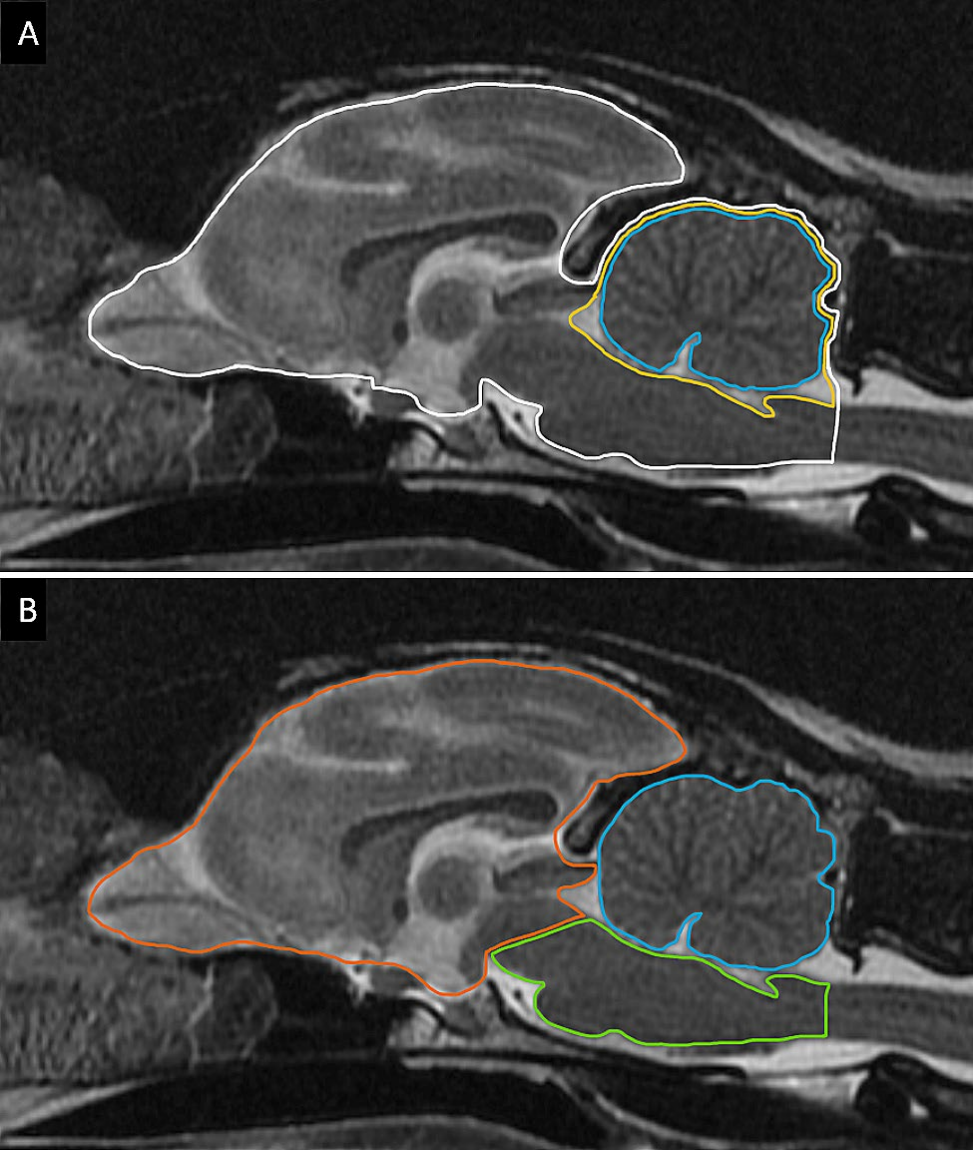
Mid-sagittal T2w FSE MRI image of an unaffected cat illustrating the methodology of MRI measurements. The measurements used were described by Henke et al. [33] and Thames et al. [32] in dogs. (**A**) Three regions of interest (ROI) depicting the entire brain (white line), cerebellum (blue line) and the cerebellum plus cerebrospinal fluid (CSF) (yellow line). The relative cerebellar size was calculated as follows: area of the cerebellum x 100/area of the entire brain. The relative CSF space was calculated as follows: (area of the cerebellum plus CSF – area of the cerebellum) x 100/ area of the cerebellum plus CSF. (**B**) Three ROI depicting the forebrain (orange line), the brainstem (green line), and the cerebellum (blue line). The brainstem: cerebellum area ratio was calculated as follows: area of the brainstem / area of the cerebellum. The cerebellum: total brain area ratio was calculated as follows: cerebellum area / (forebrain area + brainstem area + cerebellum area)

Four objective measures described by Henke et al. [33] in American Staffordshire Terrier dogs and by Thames et al. [32] in a group of different canine breeds to diagnose cerebellar cortical degeneration were measured in the three affected cats. These measures were obtained on mid-sagittal T2w FSE images, as shown in Fig. 3. The relative CSF space surrounding the cerebellum [33] was calculated as: (area of the cerebellum plus CSF – area of the cerebellum) x 100/ area of the cerebellum plus CSF (Fig. 3A). The relative cerebellar size [33] was calculated as: area of the cerebellum x 100/area of the entire brain (Fig. 3A). The cerebellum: total brain ratio [32] was calculated as: cerebellum area / (forebrain area + brainstem area + cerebellum area). Finally, the cross-sectional brainstem: cerebellum ratio [32] was calculated as: area of the brainstem / area of the cerebellum (Fig. 3B). For comparison, these measures were also done in a control group of ten adult cats (median age 8.4 years (IQR 2.1–10.3)). These control cases were selected from the most recent normal cat brain MRIs free of intracranial lesions at the CHUV. These measures are presented in Table 2, along with the established cut-offs in dogs diagnosed with cerebellar cortical degeneration.

For the relative CSF space, cases #2 and #3 had a higher relative space than the established cut-off of 12,8% in dogs and the median value of 12% measured in the group of normal cats. The results for cases #2 and #3 were suggestive of cerebellar cortical degeneration. For case #1, the least affected cat, the relative CSF space was under or within normal values of our control group and under the canine cut-off, but a progression of the relative CSF space was present between both MRI examinations.

For the relative cerebellar size and the cerebellum: total brain area ratio, all affected feline patients had a smaller result than the control group, but a higher result than the cut-offs used in dogs.

Finally, for the brainstem: cerebellum area ratio, cases #2 and #3 had a higher ratio than the control group. For the least affected cat (case #1), the ratio was within the values of our control group. All affected feline patients and our control group had a smaller ratio than the cut-off used in dogs.

**Table 2:** Comparison between published cut-offs in dogs, control cats and cats with cerebellar cortical degeneration

	Cut-off for cerebellar cortical degeneration in dogs [32, 33]	Case 1 1st MRI	Case 1 2nd MRI	Case 2	Case 3	Control cat group median (Q1-Q3)
Relative CSF space [33]	> 12,8%	8%	11,8%	20%	19%	***12%*** ***(9.75–12.5)***
Relative cerebellum size	< 13,3%	19,3%	18,1%	17,7%	16,5%	***20%*** ***(20–21.25)***
Cerebellum: total brain area ratio [32]	< 13,62%	19,5%	19%	18,9%	17%	*20.5%* *(20–21.25)*
Brainstem: cerebellum area ratio [32]	> 89%	71%	69%	78%	83%	*70%* *(66.75–76.25)*

### Gross and histopathological evaluation

**Fig. 4:**
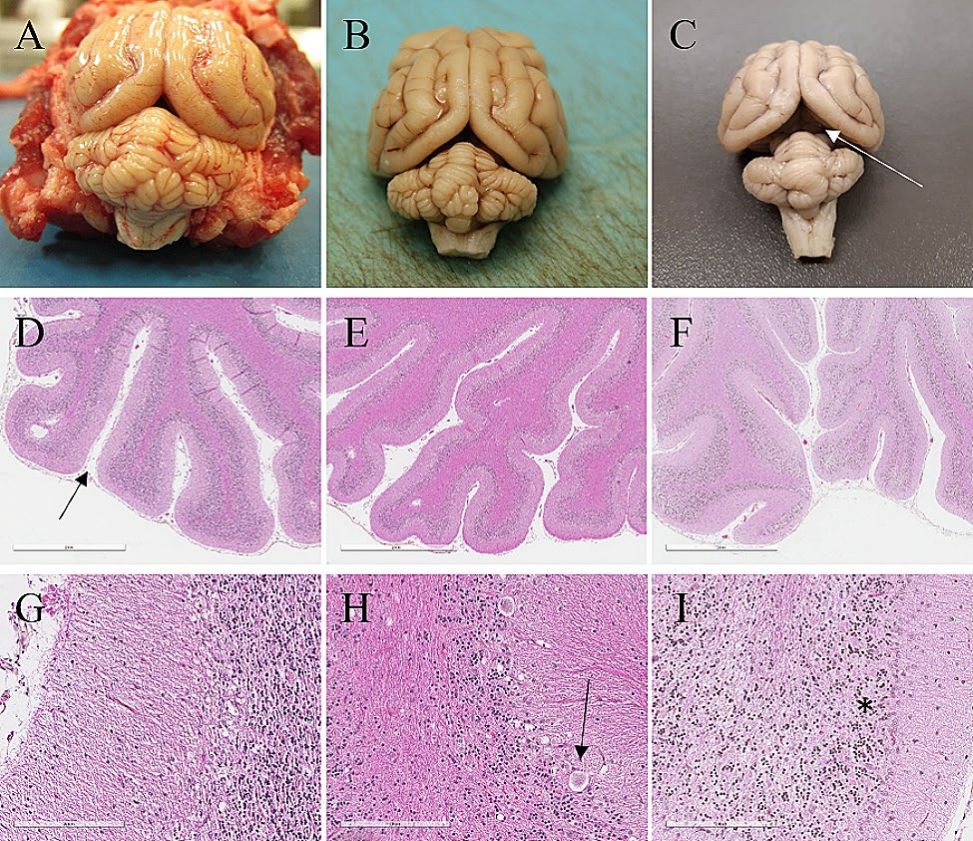
Gross and histopathological analyses of the brain/cerebellum of three cats diagnosed with cerebellar cortical degeneration. Case #1 (**A, D, G**), case #2 (**B, E, H**) and case #3 (**C, F, I**). On gross examination (**A, B, C**), there is decreased cerebellar size at different degree of severity between cases. The transverse cerebral fissure is prominent in case #3 (white arrow, C). At low magnification (**D, E, F**), histopathology revealed more prominent cerebellar sulci (black arrow, D) in all cases, along with marked pallor of cortical gray matter due to variable degrees of thinning of the granular layer. Bar = 2 mm. High magnification (**G, H, I**) shows complete loss of Purkinje cells with vacuolation and numerous empty spaces filled with eosinophilic granular material corresponding to Purkinje cell debris (black arrow, H). In the most severe case (I), the granular layer is thin, poorly cellular and pale (*). Bar = 200 μm

Gross examination of the brain in all three cases revealed varying degrees of reduction of cerebellar size as evidenced by the increased space between the brain and cerebellum (transverse cerebral fissure). The fissure was particularly prominent in case #3 (Fig. 4A, B, C). In all cases, the brain and spinal cord were collected and fixed in 10% neutral buffered formalin. Selected samples were paraffin-embedded and cut into 3 μm-thick sections for histopathological evaluation. Immunohistochemical analysis was also performed using anti-GFAP (dilution 1/500, PU020-UO, Biogenex, California, US) on slides pretreated with a standard peroxidase kit (Vectastain elite ABC, PK6100, Vector laboratories, Newark, US), revealed with AEC standard kit (AEC substrate kit, SK-4200, Vector laboratories, Newark, US) and counterstained with hematoxylin.

**Fig. 5:**
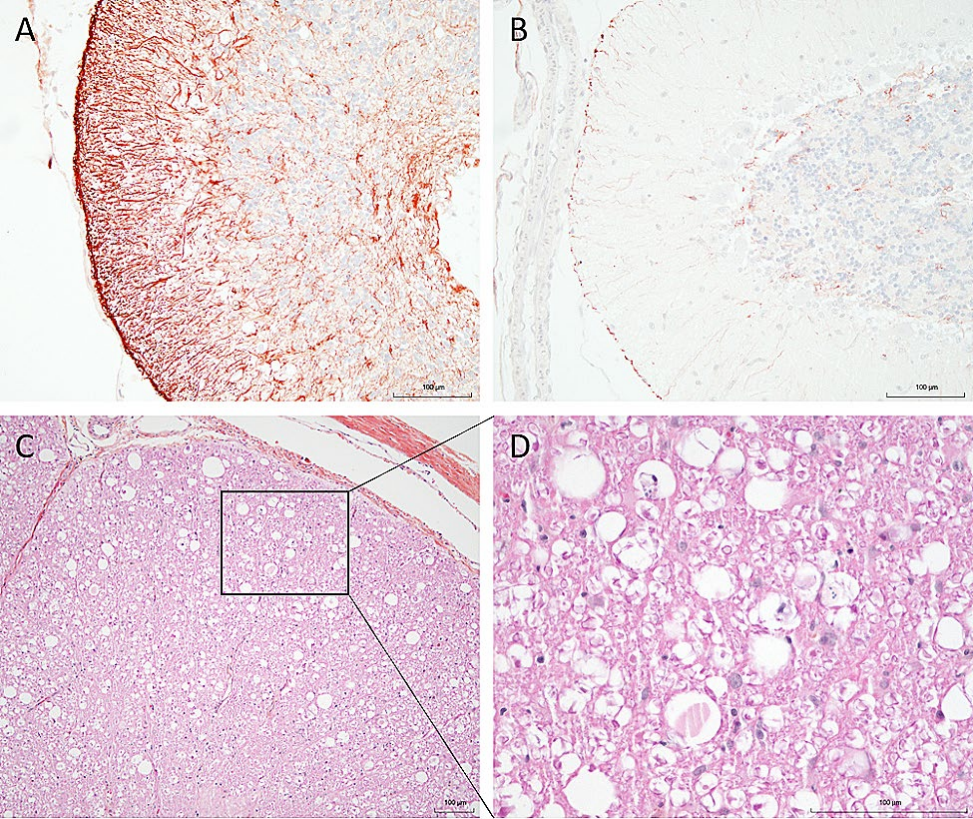
Additional histological observations. (**A, B**) Immunohistochemical analysis using anti-GFAP marker shows an increased proliferation of *Bergmann glia* (orange) in a cerebellum section of case #2, compared to a less severely affected region of the same cat (internal control) (B). (C, D) Wallerian-like degeneration noted in the dorsal spinal cord white matter of case #1 (**C**), axonal spheroids and debris, digestion chambers and empty vacuoles, visible at high magnification (**D**). Bars = 100 μm

At 25X magnification, the cerebellar sulci were widened, and the cellularity of the granular layer was variably decreased (Fig. 4D, E, F). The most prominent and consistent feature was marked to complete loss of Purkinje cells, resulting in numerous empty spaces compatible with empty baskets, along with variable vacuolation of the Purkinje cell layer (Fig. 4G, H, I). Concurrently, there was moderate to marked proliferation of *Bergmann glia*, with prominent processes radiating into the molecular layer (Fig. 5A, B). Occasional vacuolation, including digestion chambers, were observed in the cerebellar white matter. The histopathological changes were, in all cases, consistent with cerebellar cortical degeneration.

Intriguingly, in case #1, there were also degenerative lesions in the spinal cord. There was bilateral and symmetrical Wallerian-like degeneration of varying severity noted in all segments of the spinal cord (Fig. 4C, D). The lesions were more marked in and often limited to the lateral and ventral funiculi, especially the ascending spinocerebellar and other descending tracts. Additionally, chromatolysis was noted in some neurons of the dorsal root ganglia.

## Discussion and conclusions

In this case series, we described three presumably related cases of cerebellar cortical degeneration in cats and explored, for the first time, the application of MRI objective ratios as described in dogs, to facilitate the diagnostic of the disease in cats. We have shown that the relative CSF space, the relative size of the cerebellum, and the cerebellum: total brain area ratio show relevant results worth considering in future analyses. Our results further confirm post-mortem examination as the gold standard for the final diagnosis of the condition. The relation between our cases (siblings in two cases) further supports the possibility of a hereditary component in the disease. However, the presence of spinal degeneration in case #1 (not noted in cases #2 and #3) may highlight the complexity of the pathogenesis for the different forms of cerebellar cortical degeneration.

### The definition of cerebellar ataxia and clinical presentation

Cerebellar ataxia is defined as a subset of clinical ataxia involving inadequate function of the cerebellum, whether inherited or acquired. Cerebellar cortical degeneration is a cause of cerebellar ataxia and is defined as premature degeneration of fully differentiated cerebellar cells, most frequently primarily affecting Purkinje cells. There are, however, evidence of rare cases affecting primarily the granular layer both in humans [35] and animals [9]. The predominant clinical signs include difficulties in balance and gait, inability to coordinate muscle movements, and a possible lack of menace response [9]. The clinical presentation of all three cases (Table 1) showed various degree of compatible signs, without development of other intracranial nor extracranial neurological clinical signs.

### The classification of cerebellar cortical degeneration

Cerebellar cortical degeneration can be categorized into early-onset (first few weeks of age), mid-onset (first few months of age) or late-onset (adults) based on the patient’s age when the clinical signs appeared [9]. In all three feline cases presented herein, the clinical signs appeared in the first few weeks of life, classifying the degeneration as early-onset. This is in contrast with the available literature, where late-onset cases are more prevalent in dogs and cats, whereas early-onset are more common in horses [11]. Of particular interest is case #1 in which the progression of clinical signs was slower. This case also presented axonal degeneration at all levels of the spinal cord. This later change is occasionally noted in combined conditions, such as in progressive spinocerebellar degeneration [9]. This occurrence is more frequently observed in dogs, typically showing a mid- to late-onset progression of the disease compared to our cases [36]. Interestingly, only one reported case in cats had similar lesions and, like ours, presented an early-onset of the disease [8].

### Cerebellar ataxia as a genetic disorder; evidence from other species and cats

In humans, it is well established that various forms of cerebellar ataxia result from inherited disorders, and several years of investigations were required to characterize the different diseases and genes implicated [37]. While less extensively studied, there is evidence that these conditions are strongly suspected to be inherited in animals (often as autosomal recessive diseases). Notably, inherited cerebellar cortical degeneration is well-documented in Arabian horses, where a single nucleotide polymorphism variation in the MUTHY gene has been linked to the disease [19]. Subsequent experiments have delved into comprehending the different genetic alterations in this species [21].

Genetic targets have also been identified in dogs. However, there is a wide range of variations within the affected genes identified for each breed, complicating the identification of a definitive diagnostic method to prevent the transmission of the disease, although elimination of the disease in certain breeds can be successful due to its recessive nature [36]. Moreover, is it described that each type of affected genes variably influences the clinical presentation of the disease. For instance, genes associated with cerebellar cortical degeneration in dogs, including SPTBN2 [38], SEL1L [39], RAB24 [40], SNX14 [41], were shown to regulate the integrity of the cytoskeleton, protein degradation, autophagy and/or neuronal excitability [10]. On the other side, genes implicated in spinocerebellar degeneration, such as KCNJ10 [42] and SCN8A [43], play important roles in cation trafficking and mitochondrial function [10]. The pathogenesis of the different diseases may be directly related to these specific roles, hypothesizing that Purkinje cells might be more sensitive to alterations in cytoskeletal integrity and excitability, whereas the spinal cord would be more sensitive to the regulation of intracellular calcium, and therefore conductivity. In cats, substantial evidence suggests an inherited pathogenesis of the disease, although the identification of specific gene(s) remains elusive [7, 8]. Furthermore, the fact that both confirmed sibling cases (cases #1 and #2) had distinct histopathological findings, with case #1 presenting spinocerebellar degeneration concomitant to the cerebellar cortical degeneration, raises the question whether the disease may be a combination of multiple genes, or one gene with potentially large impact on both the cerebellum and spinal cord. Various techniques were considered to identify potential genes of interest in our cases. However, the associated fees for such techniques and the absence of parental material to ensure an optimal evaluation and comparison of the results prevented the execution of this plan.

### Usefulness of MRI ratios as a diagnostic ante-mortem tool, correlation between cats and dogs?

Magnetic resonance imaging stands as an indispensable ante-mortem tool for assessing cerebellar ataxia in humans [44]. Morphometric analyses and cut-offs have been proposed for certain dog breeds, most notably the American Staffordshire Terriers, and horses, specifically the Arabian horse, to aid in diagnosing the condition (Table 3) [32, 33, 45]. To our knowledge, no MRI morphometric analyses has been described in cats to date. Thus, we tried to establish normal values in a control group of cats without intracranial lesion. Considering the low sample of affected feline patients, no cut-off calculation was attempted in this report. Nevertheless, our results are interesting and show the likely usefulness of these objective measures in future cases. Indeed, all affected cases had a relative cerebellum size and a cerebellum: total brain area ratio below the values established in our control group, suggesting a decrease cerebellar size in affected cases. For the relative CSF space and the brainstem: cerebellum area ratio, only the most subjectively affected cases (cases #2 and #3) had higher values than the control group. Nevertheless, even though the least affected case (case #1) had values within our control group, an evolution of these measures was noted between both MRI examinations, suggesting a progression of the disease over time. This highlights the significance of MRI analyses in suspected cases. This underscores the notion that relying solely on a value at a single time point may prove insufficient, and if needed a follow-up examination can be useful.

It is important to note that for the relative cerebellum size and the cerebellum: total brain area ratio, and for the brainstem: cerebellum area ratio, the values of the affected cats and the control group are higher and lower, respectively, than the cut-off established in dogs for diagnosing cerebellar cortical degeneration, suggesting different morphologies between these two species. This was expected and confirmed comparing measurements with previous literature describing morphometric analyses in normal dogs, as shown in Table 4. Overall, cats seem to have a proportionally bigger cerebellum than dogs. This reinforces the need for species-specific cut-offs, and that a direct translation of the canine cut-offs cannot be used.

**Table 3:** Measures for cerebellar cortical degeneration in other species. N/A : not applicable

	Cut-offs for dogs (multiple breeds)	Cut-offs for Arabian horses
Relative CSF space	> 12,8% [33] (sensitivity 93%, specificity 100%)	> 11% [45] (sensitivity 100%, specificity 93,3%)
Relative cerebellum size	< 13,3% [33] (sensitivity 93%, specificity 94%)	< 18,9% [45] (sensitivity 60%, specificity 93,3%)
Cerebellum: total brain area ratio	< 13,62 [32] (sensitivity 96%, specificity 94%)	N/A
Brainstem: cerebellum area ratio	> 89% [32] (sensitivity 100%, specificity 100%)	N/A

**Table 4:** Magnetic resonance imaging morphometric analysis in normal dogs (literature) and cats (control group) CI : confidence interval. SD : standard deviation. N/A : not applicable

	Control group of cats Median (Q1-Q3)	Control group of cats Mean (SD; range)	Normal dogs Median (95% CI) [33]	Normal dogs Mean (SD; range) [32]
Relative CSF space	12% (9.75–12.5)	11.5% (2.7; 8.8–14.2)	9.2% (7.1–11.0%)	N/A
Relative cerebellum size	20% (20–21.25)	20.3% (1.2; 19.1–21.5)	14.9% (14.6–15.6%)	N/A
Cerebellum: total brain area ratio	20.5% (20–21.25)	20.5% (1.8; 18.7–22.3)	N/A	15.53% (1.58; 12.38–19.21)
Brainstem: cerebellum area ratio	70% (66.75–76.25)	70.8% (5.1; 65.7–75.9)	N/A	71.35% (7.11; 56.78–85.22)

### Post-mortem examination as a gold standard

Pathological changes are remarkably similar in almost all cases of cerebellar cortical degeneration. Primarily, Purkinje cells are affected, undergoing progressive degeneration and loss, while other cerebellar layers are secondarily affected, leading to phenomena such as shrinkage of granular layer and proliferation of *Bergmann glia*. Other frequently observed features include vacuolization, chromatolysis and axon degeneration within the cerebellar white matter [11]. It is important to note that the loss of Purkinje cell is not exclusive to cerebellar cortical degeneration and may be secondary to various diseases (infectious or not). In humans, multiple sclerosis is an example of such a condition. The disease is known to cause an immune-mediated demyelination of axons, which can have an impact on neuronal integrity. In the cerebellum, the local demyelination can cause variable loss of Purkinje cells, resulting in clinical cerebellar ataxia [46]. In animals, infection by viruses, such as bornaviruses [47], pestiviruses [48] and parvoviruses [4] have also been associated with Purkinje cell degeneration. In cats, parvovirus infection is of particular interest. The virus has a tropism for highly dividing cells and can affect the precursor cells of the cerebellar external granular layer *in utero* and during the perinatal period. This often results in a specific decrease in cells from the inner granular layer, usually sparring the Purkinje cells [4]. Although the different histopathological changes observed in these cases can be similar to cerebellar cortical degeneration, the preferred term for the resulting condition is cerebellar hypoplasia, as the damage caused by the virus prevents the full development of the cerebellum rather than affecting the fully differentiated cells. On a clinical standpoint, animals affected by hypoplasia often show clinical signs at birth, in opposition to the post-natal development of clinical signs for cerebellar cortical degeneration. Interestingly, some evidence suggests a possible parvoviral infection of fully differentiated neurons in young and adult cats with concomitant nervous signs, although the cases depicting this phenomenon remain scarce [49]. In our three cases, the clinical history was not compatible with infectious disease, and post-mortem evaluation did not show any histopathological evidences of infectious or inflammatory disease, and cerebellar cortical degeneration was diagnosed based on the combination of histopathological changes and clinical data.

In conclusion, our report supports a potential genetic component for cerebellar cortical degeneration in cats. Despite subjective cerebellar abnormalities on MRI examination, some MRI objective measures used for the diagnosis of this disease in other species hold promise in cats. Moreover, MRI morphometric analysis is different in dogs and cats, emphasizing the need to define feline specific cut-offs. Post-mortem evaluation of the cerebellum in combination with post-natal onset of clinical signs remain the gold standard for the final diagnosis.

## Data Availability

No datasets were generated or analysed during the current study.

## Abbreviations

CHUV: Centre Hospitalier Universitaire Vétérinaire
CSF: Cerebrospinal fluid
FELV: Feline leukemia virus
FIV: Feline immunodeficiency virus
GFAP: Glial fibrillary acidic protein
HEPS: Hematoxylin Eosin Phloxine Saffron
MRI: Magnetic resonance imaging

## References

[1] Passantino A, Masucci M (2016). Congenital and inherited neurologic diseases in dogs and cats: legislation and its effect on purchase in Italy. Veterinary World.

[2] Ueno H, Yamato O, Sugiura T, Kohyama M, Yabuki A, Miyoshi K (2016). GM1 gangliosidosis in a Japanese domestic cat: a new variant identified in Hokkaido, Japan. J Vet Med Sci.

[3] White C, Mortier J, Verin R, Maddox T, Goncalves R, Sanchez-Masian D (2018). MRI findings of neuronal ceroid lipofuscinosis in a cat. JFMS Open Rep.

[4] Résibois A, Coppens A, Poncelet L (2007). Naturally occurring parvovirus-associated feline hypogranular cerebellar hypoplasia– A comparison to experimentally-induced lesions using immunohistology. Vet Pathol.

[5] Rissi DR (2018). A retrospective study of the neuropathology and diagnosis of naturally occurring feline infectious peritonitis. J Vet Diagn Invest.

[6] Del Vecchio OV, Grande C. Toxoplasma Gondii multifocal central nervous system infiltration in an apparently immunocompetent cat in Italy. Veterinary parasitology, regional studies and reports. 2021;25:100605. 10.1016/j.vprsr.2021.100605 34474798

[7] Inada S, Mochizuki M, Izumo S, Kuriyama M, Sakamoto H, Kawasaki Y (1996). Study of hereditary cerebellar degeneration in cats. Am J Vet Res.

[8] Willoughby K, Kelly DF (2002). Hereditary cerebellar degeneration in three full sibling kittens. Vet Rec.

[9] Vandevelde M, Higgins RJ, Oevermann A (2012). Veterinary neuropathology: essentials of theory and practice.

[10] Sisó S, Hanzlícek D, Fluehmann G, Kathmann I, Tomek A, Papa V (2006). Neurodegenerative diseases in domestic animals: a comparative review. Vet J.

[11] Miller MA, Owen TJ, Bruyette DS, Scott-Moncrieff JC, Ramos-Vara JA, Weng HY (2018). Immunohistochemical evaluation of Canine Pituitary Adenomas obtained by Transsphenoidal Hypophysectomy. Vet Pathol.

[12] Wade CM, Pan AYH, Taylor RM, Williamson P. Cerebellar Abiotrophy in Australian Working Kelpies is Associated with two major Risk Loci. Genes (Basel). 2022;13(10). 10.3390/genes13101709 PMC9602046 36292596

[13] Bertalan A, Glass EN, Kent M, De LaHunta A, Bradley C (2014). Late-onset cerebellar abiotrophy in a Labrador Retriever. Aust Vet J.

[14] Sen C, Sharma AK, Randhawa CS, Gupta K (2017). Cerebellar cortical Abiotrophy in Young Labrador-Retrievers. Top Companion Anim Med.

[15] Berry ML, Blas-Machado U (2003). Cerebellar abiotrophy in a miniature schnauzer. Can Vet J.

[16] Sandy JR, Slocombe RE, Mitten RW, Jedwab D (2002). Cerebellar abiotrophy in a family of Border Collie dogs. Vet Pathol.

[17] Kent M, Glass E, deLahunta A (2000). Cerebellar cortical abiotrophy in a beagle. J Small Anim Pract.

[18] van Tongern SE, van Vonderen IK, van Nes JJ, van den Ingh TS (2000). Cerebellar cortical abiotrophy in two Portuguese podenco littermates. Vet Q.

[19] Scott EY, Woolard KD, Finno CJ, Penedo MCT, Murray JD (2018). Variation in MUTYH expression in arabian horses with cerebellar Abiotrophy. Brain Res.

[20] Sadaba SA, Madariaga GJ, Botto CM, Carino MH, Zappa ME, García PP (2016). First report of cerebellar abiotrophy in an arabian foal from Argentina. Open Vet J.

[21] Scott EY, Penedo MCT, Murray JD, Finno CJ (2017). Defining trends in Global Gene expression in arabian horses with cerebellar Abiotrophy. Cerebellum.

[22] Cavalleri JMV, Metzger J, Hellige M, Lampe V, Stuckenschneider K, Tipold A (2013). Morphometric magnetic resonance imaging and genetic testing in cerebellar abiotrophy in arabian horses. BMC Vet Res.

[23] Blanco A, Moyano R, Vivo J, Flores-Acuña R, Molina A, Blanco C (2006). Purkinje cell apoptosis in arabian horses with cerebellar abiotrophy. J Vet Med Physiol Pathol Clin Med.

[24] Koehler JW, Newcomer BW, Holland M, Caldwell JM (2015). A novel inherited cerebellar Abiotrophy in a cohort of related goats. J Comp Pathol.

[25] Milne EM, Schock A (1998). Cerebellar abiotrophy in a pedigree Charollais sheep flock. Vet Rec.

[26] Sato J, Sasaki S, Yamada N, Tsuchitani M (2011). Hereditary Cerebellar degenerative disease (cerebellar cortical abiotrophy) in rabbits. Vet Pathol.

[27] Kemp J, McOrist S, Jeffrey M (1995). Cerebellar abiotrophy in Holstein Friesian calves. Vet Rec.

[28] Biolatti C, Gianella P, Capucchio MT, Borrelli A, D’Angelo A (2010). Late onset and rapid progression of cerebellar abiotrophy in a domestic shorthair cat. J Small Anim Pract.

[29] Negrin A, Bernardini M, Baumgärtner W, Castagnaro M (2006). Late onset cerebellar degeneration in a middle-aged cat. J Feline Med Surg.

[30] Barone G, Foureman P, deLahunta A (2002). Adult-onset cerebellar cortical abiotrophy and retinal degeneration in a domestic shorthair cat. J Am Anim Hosp Assoc.

[31] Shamir M, Perl S, Sharon L (1999). Late onset of cerebellar abiotrophy in a siamese cat. J Small Anim Pract.

[32] Thames RA, Robertson ID, Flegel T, Henke D, O’Brien DP, Coates JR (2010). Development of a morphometric magnetic resonance image parameter suitable for distinguishing between normal dogs and dogs with cerebellar atrophy. Vet Radiol Ultrasound.

[33] Henke D, Böttcher P, Doherr MG, Oechtering G, Flegel T (2008). Computer-assisted magnetic resonance imaging brain morphometry in American Staffordshire Terriers with cerebellar cortical degeneration. J Vet Intern Med.

[34] Woodard JC, Collins GH, Hessler JR (1974). Feline hereditary neuroaxonal dystrophy. Am J Pathol.

[35] Pascual-Castroviejo I, Gutierrez M, Morales C, Gonzalez-Mediero I, Martínez-Bermejo A, Pascual-Pascual SI (1994). Primary degeneration of the granular layer of the cerebellum. A study of 14 patients and review of the literature. Neuropediatrics.

[36] Stee K, Van Poucke M, Lowrie M, Van Ham L, Peelman L, Olby N (2023). Phenotypic and genetic aspects of hereditary ataxia in dogs. J Vet Intern Med.

[37] Coarelli G, Wirth T, Tranchant C, Koenig M, Durr A, Anheim M (2023). The inherited cerebellar ataxias: an update. J Neurol.

[38] Forman OP, De Risio L, Stewart J, Mellersh CS, Beltran E (2012). Genome-wide mRNA sequencing of a single canine cerebellar cortical degeneration case leads to the identification of a disease associated SPTBN2 mutation. BMC Genet.

[39] Kyöstilä K, Cizinauskas S, Seppälä EH, Suhonen E, Jeserevics J, Sukura A (2012). A SEL1L mutation links a canine progressive early-onset cerebellar ataxia to the endoplasmic reticulum-associated protein degradation (ERAD) machinery. PLoS Genet.

[40] Agler C, Nielsen DM, Urkasemsin G, Singleton A, Tonomura N, Sigurdsson S (2014). Canine hereditary ataxia in old English sheepdogs and gordon setters is associated with a defect in the autophagy gene encoding RAB24. PLoS Genet.

[41] Fenn J, Boursnell M, Hitti RJ, Jenkins CA, Terry RL, Priestnall SL (2016). Genome sequencing reveals a splice donor site mutation in the SNX14 gene associated with a novel cerebellar cortical degeneration in the Hungarian Vizsla dog breed. BMC Genet.

[42] Gilliam D, O’Brien DP, Coates JR, Johnson GS, Johnson GC, Mhlanga-Mutangadura T (2014). A homozygous KCNJ10 mutation in Jack Russell Terriers and related breeds with spinocerebellar ataxia with myokymia, seizures, or both. J Vet Intern Med.

[43] Letko A, Dietschi E, Nieburg M, Jagannathan V, Gurtner C, Oevermann A et al. A missense variant in SCN8A in Alpine Dachsbracke Dogs affected by Spinocerebellar Ataxia. Genes (Basel). 2019;10(5). 10.3390/genes10050362 PMC6562999 31083464

[44] Cocozza S, Pontillo G, De Michele G, Di Stasi M, Guerriero E, Perillo T (2021). Conventional MRI findings in hereditary degenerative ataxias: a pictorial review. Neuroradiology.

[45] Cavalleri JM, Metzger J, Hellige M, Lampe V, Stuckenschneider K, Tipold A (2013). Morphometric magnetic resonance imaging and genetic testing in cerebellar abiotrophy in arabian horses. BMC Vet Res.

[46] Redondo J, Kemp K, Hares K, Rice C, Scolding N, Wilkins A (2015). Purkinje Cell Pathology and loss in multiple sclerosis cerebellum. Brain Pathol.

[47] Williams BL, Yaddanapudi K, Hornig M, Lipkin WI (2007). Spatiotemporal analysis of purkinje cell degeneration relative to parasagittal expression domains in a model of neonatal viral infection. J Virol.

[48] Toplu N, Oğuzoğlu TÇ, Epikmen ET, Aydoğan A (2010). Neuropathologic study of Border Disease Virus in naturally infected fetal and neonatal small ruminants and its Association with apoptosis. Vet Pathol.

[49] Garigliany M, Gilliaux G, Jolly S, Casanova T, Bayrou C, Gommeren K (2016). Feline panleukopenia virus in cerebral neurons of young and adult cats. BMC Vet Res.

